# Severe spruelike enteropathy and collagenous colitis caused by olmesartan

**DOI:** 10.1186/s12876-021-01926-y

**Published:** 2021-09-23

**Authors:** Shiho Kaneko, Kana Matsuda, Yasuko Mizuta, Shoya Shiratori, Kazuma Kishi, Akihisa Nakamura, Masataka Yagisawa, Nobuyuki Ehira, Minoru Uebayashi, Hiroya Kobayashi

**Affiliations:** 1 Depertment of Gastroenterology, Kitami Red Cross Hospital, Higashi-2, Kita-6, Kitami, 090-0026 Japan; 2 Department of Pathology, Asahikawa Medical University, 1-1 Midorigaoka Higashi-2 Hokkaido, Asahikawa, Japan

**Keywords:** Villous atrophy, Collagenous colitis, Spruelike enteropathy, Intestinal diseases, Case report

## Abstract

**Background:**

Olmesartan, which is an angiotensin II receptor blocker, reportedly causes spruelike enteropathy, with intestinal villous atrophy as its typical histopathological finding. Interestingly, collagenous and/or lymphocytic gastritis and colitis occur in some patients. We report the case of a 73-year-old Japanese man with a 2-month clinical history of severe diarrhea and weight loss. There were few reports in which spruelike enteropathy and collagenous colitis were both observed and could be followed up.

**Case presentation:**

We report a case of a 73-year-old man with a 2-month clinical history of severe diarrhea and weight loss. He had taken olmesartan for hypertension treatment for 5 years. Endoscopic examination with biopsies revealed intestinal villous atrophy and collagenous colitis. Suspecting enteropathy caused by olmesartan, which was discontinued on admission because of hypotension, we continued to stop the drug. Within 3 weeks after olmesartan discontinuation, his clinical symptoms improved. After 3 months, follow-up endoscopy showed improvement of villous atrophy but not of the thickened collagen band of the colon. However, the mucosa normalized after 6 months, histologically confirming that the preexistent pathology was finally resolved.

**Conclusions:**

This report presents a case in which spruelike enteropathy and collagenous colitis were both observed and could be followed up. In unexplained cases of diarrhea, medication history should be reconfirmed and this disease should be considered a differential diagnosis.

**Supplementary Information:**

The online version contains supplementary material available at 10.1186/s12876-021-01926-y.

## Background

,^1^,^1^,^1^,^2^]. Pathological evidence of the stomach and colon suggests that olmesartan may affect the entire gastrointestinal tract. There were few reports in which spruelike enteropathy and collagenous colitis were both observed and could be followed up.

Herein, we report a case of severe enteropathy and collagenous colitis associated with olmesartan use.

## Case presentation

**Fig. 1:**
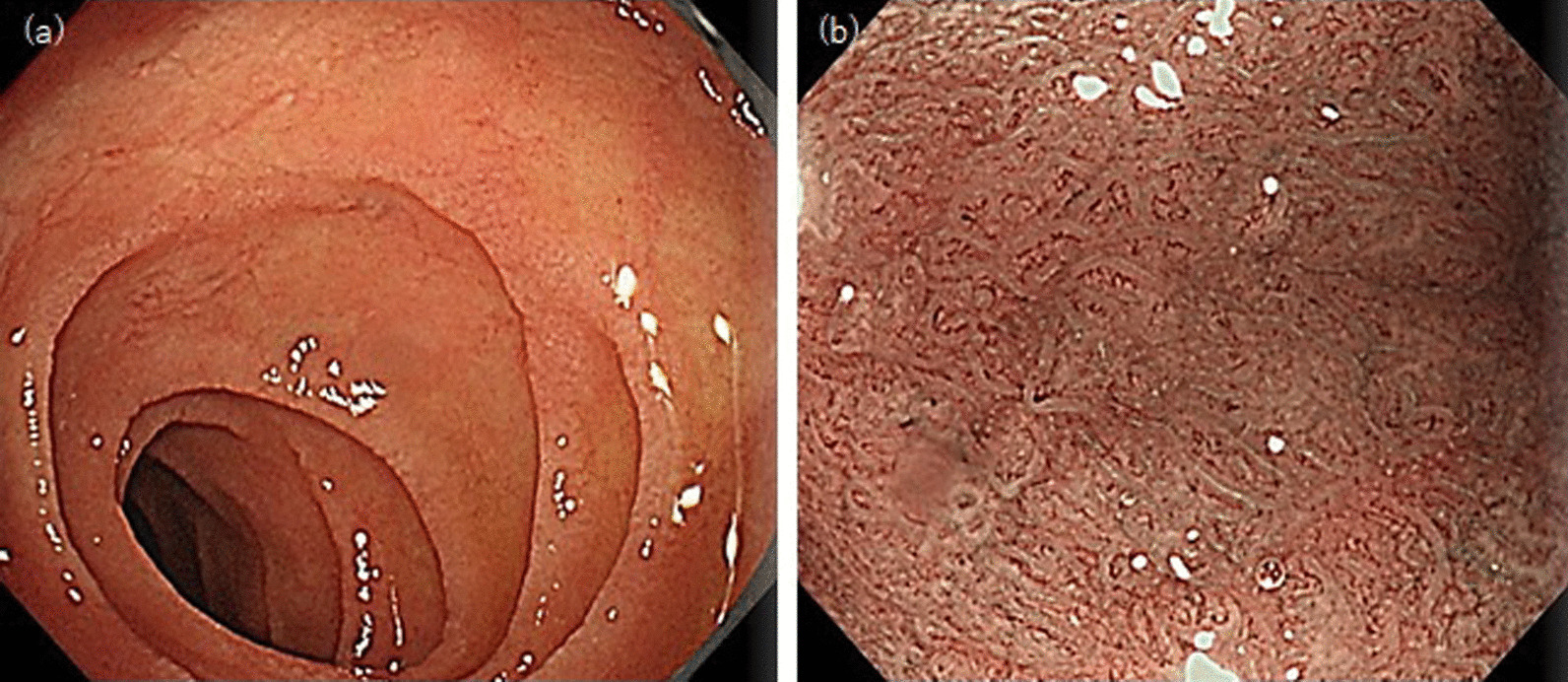
Esophagogastroduodenoscopy at the initial visit. **a** White-light imaging shows villous atrophy and a mosaic pattern of the duodenal mucosa. **b** Magnification endoscopy with narrow-band imaging shows villous atrophy of the duodenal mucosa

**Fig. 2:**
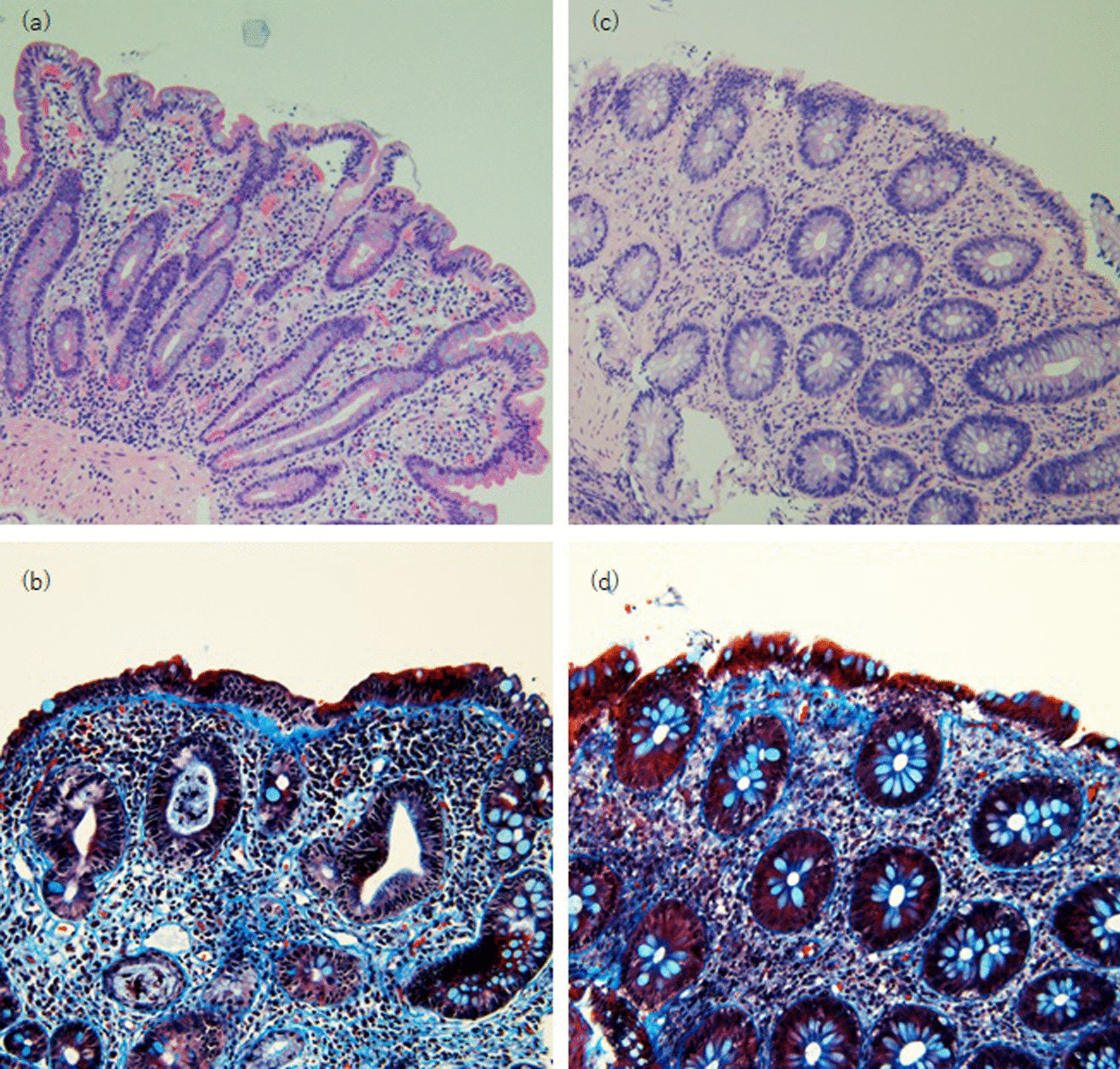
**a** Duodenal biopsy (hematoxylin–eosin, 0924; top: –743; width: 5205; height: 3719; visibility: visible; mso-wrap-style: square); **b** Duodenal biopsy (Masson trichrome, ichromeichrome eosin, 0924; top: Colonic biopsy (hematoxylin–eosin, 0924; top: − 743; width: 5205; height: 3719; visibility: visible)

A 73-year-old Japanese man with a history of olmesartan intake (20 mg daily for 5 years) for hypertension treatment was admitted to a local hospital complaining of watery, nonbloody diarrhea approximately 10 times daily since 2 months. In 2 months, he lost 10 kg of his weight. Blood tests, CT, and endoscopy were performed, but the cause of his diarrhea remained unknown. Hence, he was referred to our hospital for further examination. His physical examination results were unremarkable. However, laboratory results (Additional file 1) indicated anemia (hemoglobin, 10.5 g/dL) and hypoalbuminemia (3.4 g/dL). We searched DQA1 and DQB1. HLA DQ4 and DQ6 were positive. Meanwhile, stool culture, Clostridium difficile toxin, HLA-DQ2/DQ8, and IgA antibodies to tissue transglutaminase and endomysial, were all negative. Abdominal computer tomography was unremarkable. Esophagogastroduodenoscopy (EGD) revealed villous atrophy and a mosaic pattern of the duodenal mucosa (Fig. 1), while colonoscopy (CS) detected villous atrophy of the terminal ileum and diffuse slight edema of the colon (Additional file 2). The stomach, duodenum, terminal ileum, and colon were randomly biopsied. Pathological findings of the duodenum and ileum showed villous atrophy, intraepithelial lymphocyte infiltration, and collagen band, and those of the colon showed a 14 μm collagen band (Fig. 2). Moreover, capsule endoscopy displayed villous atrophy of the entire small intestine. Taken together, we suspected that the patient had olmesartan-associated spruelike enteropathy. Hence, olmesartan, which was stopped on admission because of hypotension, remained withdrawn, and was switched to amlodipine.

**Fig. 3:**
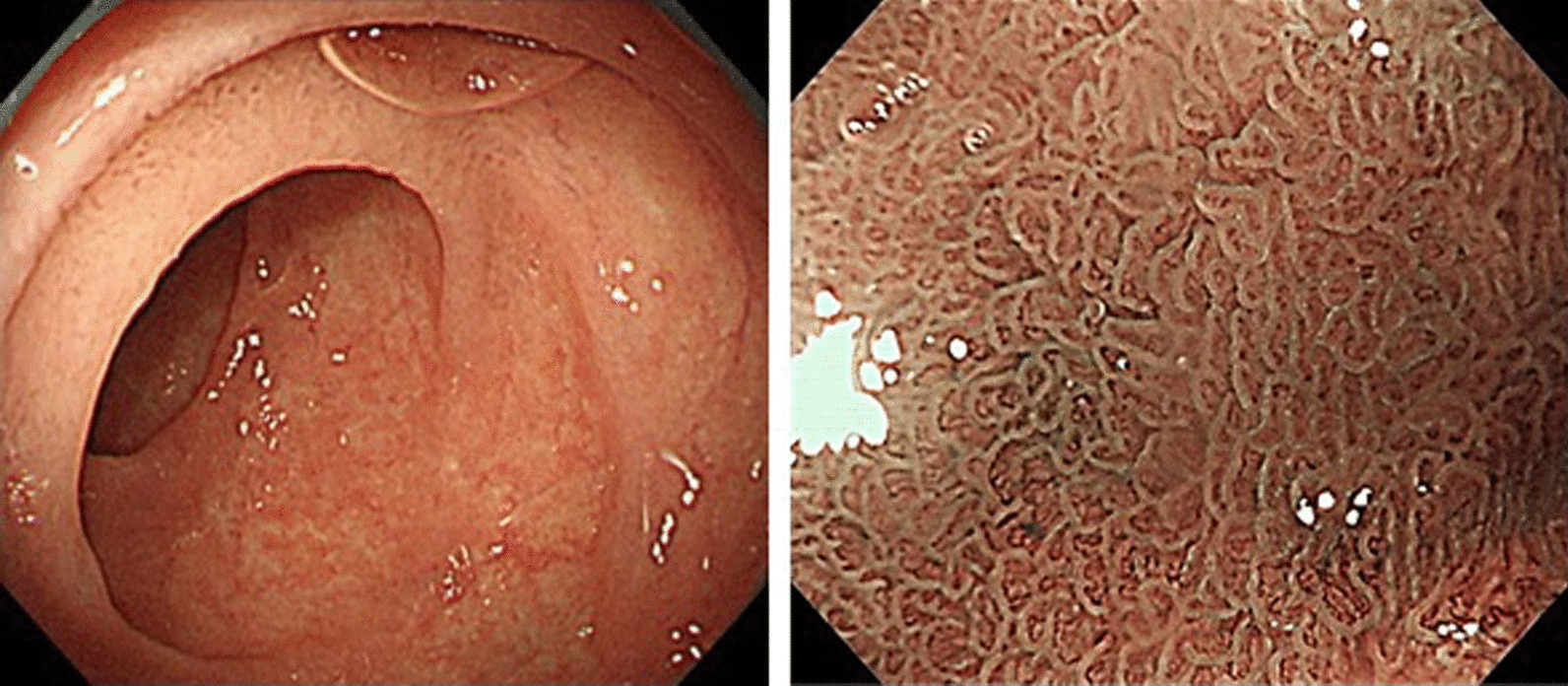
Esophagogastroduodenoscopy showed a normal appearance of the duodenum 3 months after olmesartan discontinuation

**Fig. 4:**
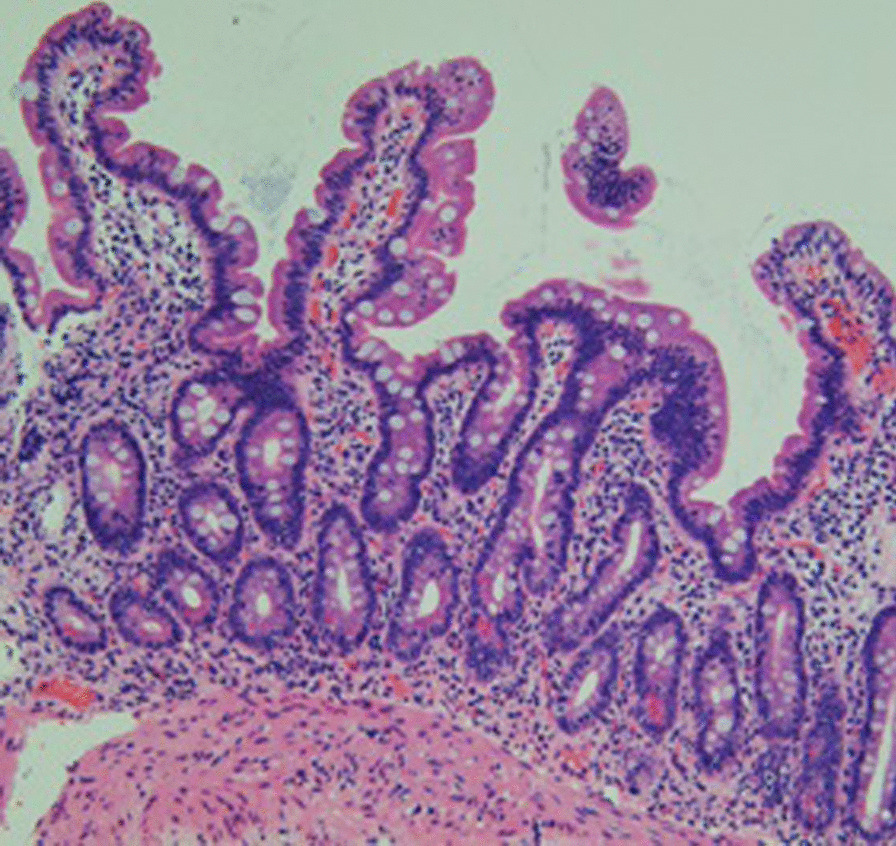
Biopsy showed an almost complete recovery of the villi on the duodenal mucosa 3 months after olmesartan discontinuation (hematoxylin–eosin, redof)

Within 3 weeks after olmesartan discontinuation, his clinical symptoms improved. Three months later, diarrhea resolved, and the duodenum, terminal ileum, and colon showed a normal appearance on EGD/CS (Fig. 3). As shown in the biopsies of the duodenum and terminal ileum, the villous architecture of the duodenal and ileal mucosa almost completely recovered (Fig. 4), but in colonic biopsies, the collagen band only slightly improved (Additional file 3). Six months after discontinuing olmesartan, follow-up endoscopy showed a histologically normal colonic mucosa.

## Discussion and conclusion

Olmesartan-associated spruelike enteropathy is a type of enteropathy induced by olmesartan administration. It is characterized by severe diarrhea and weight loss. The duration of olmesartan exposure before the onset of diarrhea varies from months to years [1,3,1,1,1,4]. HLA-DQ2 or DQ8 haplotypes were present in 71% of patients with olmesartan-associated spruelike enteropathy [3,1,5,6,2,

,^2^]. CS or capsule endoscopy may reveal villous atrophy and ulceration in the jejunum and ileum [2,

,^7^,^8^,^9^,

,^1^,^10^,^11^,^12^,

,^13^,^14^,^1^,^15^,^16^,^17^,^15^]. Some patients with collagenous colitis showed a typical histology but had no symptoms [^18^,

Hence, this report presents a rare case in which spruelike enteropathy and collagenous colitis were both observed and could be followed up. Villous atrophy of the small intestine may have a greater effect on diarrhea caused by olmesartan even in cases with collagenous colitis.

In conclusion, both the large and small intestines should be assessed for villous atrophy and other abnormalities.

## Supplementary Information

**Additional file 1.** Initial Laboratory Studies.

**Additional file 2.** Colonoscopy showed a diffuse slight edema of the colon.

**Additional file 3.** Biopsy showed slight improvement of the collagen band in the colon 3 months after olmesartan discontinuation (Masson trichrome).

## Data Availability

The datasets used and/or analyzed during the current study are available from the corresponding author on reasonable request.

## Abbreviations

ARBs: Angiotensin II receptor blockers
CS: Colonoscopy
EGD: Esophagogastroduodenoscopy
