# Supplementary material for: Severe spruelike enteropathy and collagenous colitis caused by olmesartan
Source: BMC Gastroenterol. 2021 Sep 23;21:350. doi: 10.1186/s12876-021-01926-y (PMC8461977; doi:10.1186/s12876-021-01926-y)
Supplement: Supplementary file 1 — Additional file 1. Initial Laboratory Studies. [file 12876_2021_1926_MOESM1_ESM.docx]

Supplementary Material 1. Initial Laboratory Studies

| WBC | 4,150 | /mm^3^ |
| --- | --- | --- |
| Neu | 67.5 | % |
| Lym | 21.9 | % |
| RBC | 317 | million/mm^3^ |
| Hb | 10.5 | g/dL |
| Ht | 31.9 | % |
| Plt | 18.1 | million/mm^3^ |
| T-Bil | 0.3 | mg/dL |
| TP | 5.5 | g/dL |
| Alb | 3.4 | g/dL |
| AST | 35 | U/L |
| ALT | 41 | U/L |
| LDH | 228 | U/L |
| ALP | 244 | U/L |
| γGTP | 32 | U/L |
| Amy | 128 | U/L |
| BUN | 7.8 | mg/dL |
| Cr | 0.62 | mg/dL |
| Na | 149 | mEq/L |
| K | 3.4 | mEq/L |
| Cl | 115 | mEq/L |
| CRP | 0.06 | mg/dL |
| TSH | 2.25 | μU/mL |
| FT4 | 1.03 | ng/dL |
| CEA | 5.0 | ng/mL |
| CA19-9 | 12.1 | U/mL |
