# Supplementary figures and images for: Severe spruelike enteropathy and collagenous colitis caused by olmesartan
Source: BMC Gastroenterol. 2021 Sep 23;21:350. doi: 10.1186/s12876-021-01926-y (PMC8461977; doi:10.1186/s12876-021-01926-y)

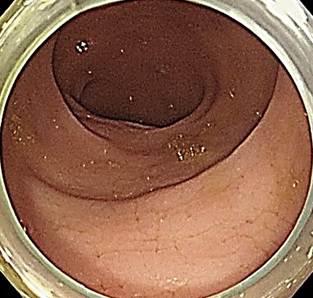

Supplement: Supplementary file 2 — Additional file 2. Colonoscopy showed a diffuse slight edema of the colon. [file 12876_2021_1926_MOESM2_ESM.jpg]

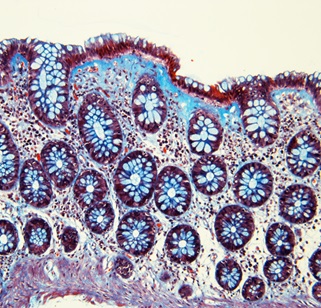

Supplement: Supplementary file 3 — Additional file 3. Biopsy showed slight improvement of the collagen band in the colon 3 months after olmesartan discontinuation (Masson trichrome). [file 12876_2021_1926_MOESM3_ESM.jpg]
